# Protective Effects of 2,3,5,4′-Tetrahydroxystilbene-2-*O*-β-d-glucoside on Ovariectomy Induced Osteoporosis Mouse Model

**DOI:** 10.3390/ijms19092554

**Published:** 2018-08-28

**Authors:** Su-Jin Kim, Yun-Ho Hwang, Seul-Ki Mun, Seong-Gyeol Hong, Kwang-Jin Kim, Kyung-Yun Kang, Young-Jin Son, Sung-Tae Yee

**Affiliations:** 1 Department of Pharmacy, Sunchon National University, 255 Jungangno, Suncheon 540-950, Korea; ksz1353@naver.com (S.-J.K.); hyh7733@naver.com (Y.-H.H.); motomoto1210@naver.com (S.-K.M.); hong9217@naver.com (S.-G.H.); mastiffk@naver.com (K.-J.K.); sony@sunchon.ac.kr (Y.-J.S.); 2 Suncheon Research Center for Natural Medicines, Suncheon 540-950, Korea; kang8404@nate.com

**Keywords:** 2,3,5,4′-tetrahydroxystilbene-2-*O*-β-d-glucoside (TSG), osteoporosis, ovariectomy, bone loss, menopause

## Abstract

2,3,5,4′-Tetrahydroxystilbene-2-*O*-β-d-glucoside (TSG), an active polyphenolic component of *Polygonum multiflorum*, exhibits many pharmacological activities including antioxidant, anti-inflammation, and anti-aging effects. A previous study demonstrated that TSG protected MC3T3-E1 cells from hydrogen peroxide (H_2_O_2_) induced cell damage and the inhibition of osteoblastic differentiation. However, no studies have investigated the prevention of ovariectomy-induced bone loss in mice. Therefore, we investigated the effects of TSG on bone loss in ovariectomized mice (OVX). Treatment with TSG (1 and 3 μg/g; i.p.) for six weeks positively affected body weight, uterine weight, organ weight, bone length, and weight change because of estrogen deficiency. The levels of the serum biochemical markers of calcium (Ca), inorganic phosphorus (IP), alkaline phosphatase (ALP), and total cholesterol (TCHO) decreased in the TSG-treated mice when compared with the OVX mice. Additionally, the serum bone alkaline phosphatase (BALP) levels in the TSG-treated OVX mice were significantly increased compared with the OVX mice, while the tartrate-resistant acid phosphatase (TRAP) activity was significantly reduced. Furthermore, the OVX mice treated with TSG showed a significantly reduced bone loss compared to the untreated OVX mice upon micro-computed tomography (CT) analysis. Consequently, bone destruction in osteoporotic mice as a result of ovariectomy was inhibited by the administration of TSG. These findings indicate that TSG effectively prevents bone loss in OVX mice; therefore, it can be considered as a potential therapeutic for the treatment of postmenopausal osteoporosis.

## 1. Introduction

Osteoporosis is characterized by decreased bone mass and bone microstructure destruction, which increases the risk of fracture. According to U.S. statistics, approximately 30% of postmenopausal women experience osteoporotic fractures, resulting in direct and indirect costs of approximately U.S. $10 billion per year [1].

Osteoporosis normally occurs more often in women than men [2], especially after menopause, when bone loss rapidly increases [3]. The average age of Korean menopause is 51 years, and it is primarily caused by a reduction of female hormones due to ovarian dysfunction [4,5]. Estrogen, a hormone secreted by the ovary, plays a role in the inhibition of bone resorption and has significant effects on bone formation. Specifically, blood estrogen inhibits the destruction of bones via osteoclast differentiation, promotes the differentiation of osteoblasts that make bone, and protects bone by maintaining osteogenesis [6,7].

Osteoporosis medication is largely divided into bone resorption inhibitors and bone formation promoters. Bisphosphonates, selective estrogen receptor modulators (SERMs), and hormone replacement therapies (HRT) are used as bone resorption inhibitors, while the parathyroid hormone is used as an osteogenesis stimulant [8]. Bisphosphonate is currently the most widely used therapeutic agent for the prevention and treatment of postmenopausal osteoporosis [9,10]. Selective estrogen receptor modulators are similar to estrogen, and SERM raloxifene increases the bone mineral density and reduces the risk of spinal fractures [11]. Hormone replacement therapy to supplement female hormone deficiency, caused by menopause, is mainly used in combination with estrogen. For example, estrogen plus progesterone is commonly used to protect bones and the cardiovascular system [12]. However, the side effects of osteoporosis medication have been reported. For example, bisphosphonates, which inhibit bone resorption, can cause side effects such as jawbone necrosis, atypical femoral fractures, and serious complications of bone necrosis [13,14]. In addition, selective estrogen receptor modulators increase the incidence of high flush, venous thromboembolism (VTE), and pulmonary embolism, and hormone replacement therapy (HRT) has side effects that include an increased risk of breast cancer, stroke, and coronary artery disease [15,16,17,18]. Furthermore, the administration of parathyroid hormone (PTH) to osteoporosis patients induces adverse effects such as headache, nausea, cramps, and hypercalcemia [19].

Recently, the use of natural materials, with few side effects, has been studied in order to overcome the aforementioned problems. An active component, 2,3,5,4′-tetrahydroxystilbene-2-*O*-β-d-glucoside (TSG), isolated from *Polygonum multiflorum*, demonstrated that has many pharmacological activities, such as antioxidative and free radical-scavenging properties [20,21]. Moreover, TSG reduces hyperlipidemia, prevents lipid peroxidation, and protects the cardiovascular system [22,23]. In addition, TSG has anti-inflammatory, anti-aging, and melanogenesis activities, while also improving cardiac function and preventing atherosclerosis [24,25,26,27,28]. Moreover, TSG was found to exert protective effects against diabetic nephropathy in the mice with hyperglycemia induced by streptozotocin [29], and was recently shown to effectively prevent apoptosis induced hair loss, alleviate the development of periodontitis, and protect MC3T3-E1 cells from H_2_O_2_-induced cell damage and the inhibition of osteoblastic differentiation [30,31,32].

Although TSG has been shown to have anti-osteoarthritis activities through in vitro and mono-iodoacetate inductive models [33], no studies have shown that it effectively prevents the bone loss caused by the ovarian deficiency in ovariectomized mice. Therefore, this study was conducted to determine whether TSG effectively prevents bone loss in the ovariectomized (OVX) mice, and if it can be considered a potential therapeutic for the treatment of postmenopausal osteoporosis.

## 2. Results

### 2.1. Effects of TSG on Body Weight of Ovariectomized (OVX) Mice

**Figure 1:**
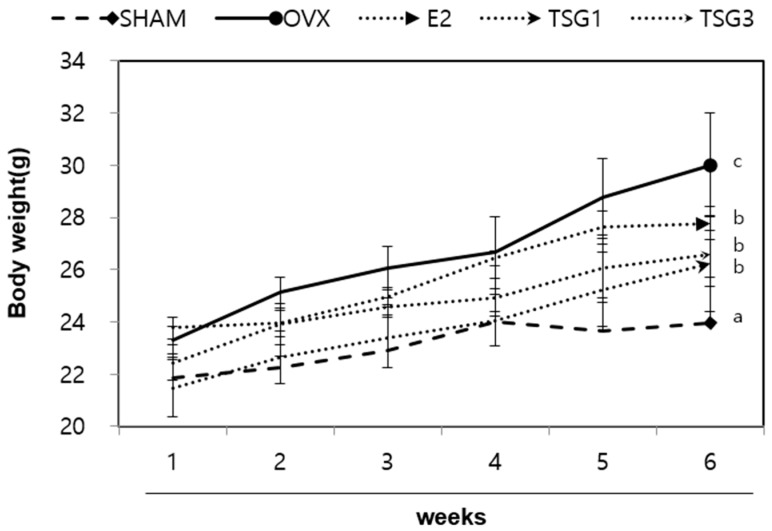
Effect of TSG on body weight. Body weights were measured at weekly intervals and the effect of TSG was seen at six weeks. a, b, and c: The means not sharing a common letter are significantly different among group at *p* < 0.05 by one-way analysis of variance (ANOVA) with Duncan’s multiple-range test. SHAM (sham-operated control group); OVX (ovariectomized group); TSG1 (1 μg OVX TSG group); TSG 3 (3 μg OVX TSG group).

The weights of the experimental animals were measured at weekly intervals in order to confirm the change in weight after menopause. There were no differences in the initial weights of the five groups; however, the body weight of the OVX group was significantly increased compared to that of the SHAM (sham-operated control) group after six weeks. In addition, the body weight of the OVX+TSG (1 and 3 μg/g) groups decreased significantly compared with the weight of the OVX group. These findings demonstrated that TSG has an inhibitory effect on the increase in weight induced by estrogen deficiency (Figure 1).

### 2.2. Effect of TSG on Organ (Uterus, Spleen, and Thymus) Weight in OVX Mice

**Figure 2:**
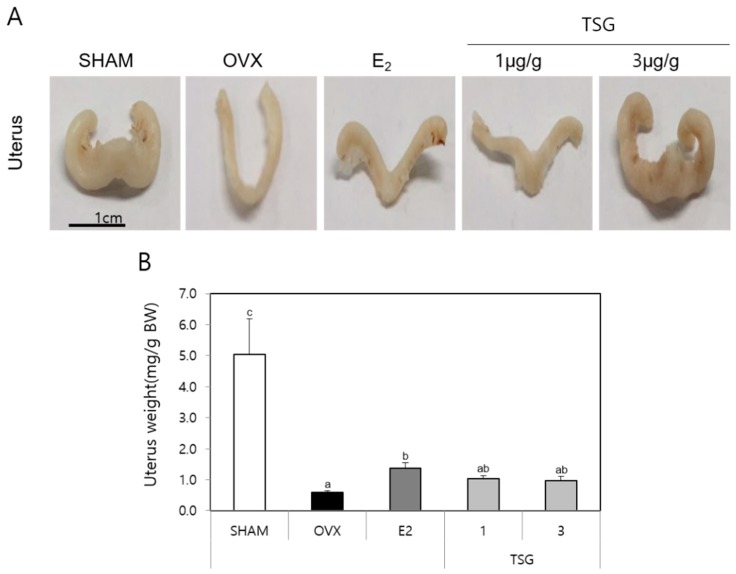
Effect of TSG on uterus weight. The C3H/HeN mice were treated with TSG for 6 weeks, and the uteri were harvested 24 h after the last treatment. (**A**) The uterus was photographed with a digital camera and (**B**) weighed. a, b, and c: The means not sharing a common letter are significantly different among group at *p* < 0.05 by one-way ANOVA with Duncan’s multiple-range test.

After being sacrificed, the size of the uterus was evaluated using a digital camera, which revealed that the OVX group’s uterus size decreased compared with the SHAM group (Figure 2A). Moreover, the OVX+TSG treated groups showed a greater increase in the uterus size than the OVX group. The exact uterine changes were confirmed by weighing the uterus (Figure 2B). The OVX group uterus weight decreased compared with the SHAM group. In addition, the uterus weights of the OVX+TSG (1 and 3 μg/g) treated groups increased relative to the OVX group, but this difference was not significant. Taken together, these results demonstrated that TSG protected against the decreased uterus weight caused by estrogen deficiency. The thymus and spleen are representative immune organs associated with estrogen; therefore, we evaluated the effects of TSG on their weights (Table 1). The weights of the thymus and spleen of the OVX group increased relative to the SHAM group, but this increase was not significant. However, the thymus weights of the OVX+TSG groups (1 and 3 µg/g) were significantly lower than those of the OVX group. Moreover, the spleen weight of the OVX+TSG (1 and 3 µg/g) group decreased significantly, relative to the OVX group. These findings demonstrated that TSG has an inhibitory effect on the decreased thymus and spleen weight by estrogen deficiency.

### 2.3. Effect of TSG on Bone Weight and Length in OVX Mice

After the animal sacrifice, the weight and length of the bones were measured using a Vernier caliper and an electronic scale. The length and weight of the tibia in the OVX group were significantly lower than in the SHAM group. The weight of the femur in the OVX group was significantly lower than in the SHAM group. However, the tibia length of the OVX+TSG (1 and 3 μg/g) group were significantly longer than that of the OVX group. Moreover, the tibia weights of the OVX+TSG (1 and 3 μg/g) groups were significantly increased compared with the OVX group, and the femur weight of the OVX+TSG (3 μg/g) group was increased significantly compared with the OVX group. These results suggest that TSG induces bone growth (Table 2).

### 2.4. Effect of TSG on Serum Biochemical Markers (Calcium (Ca), Inorganic Phosphorus (IP), Alkaline Phosphatase (ALP), and Total Cholesterol (TCHO)) in OVX Mice

**Figure 3:**
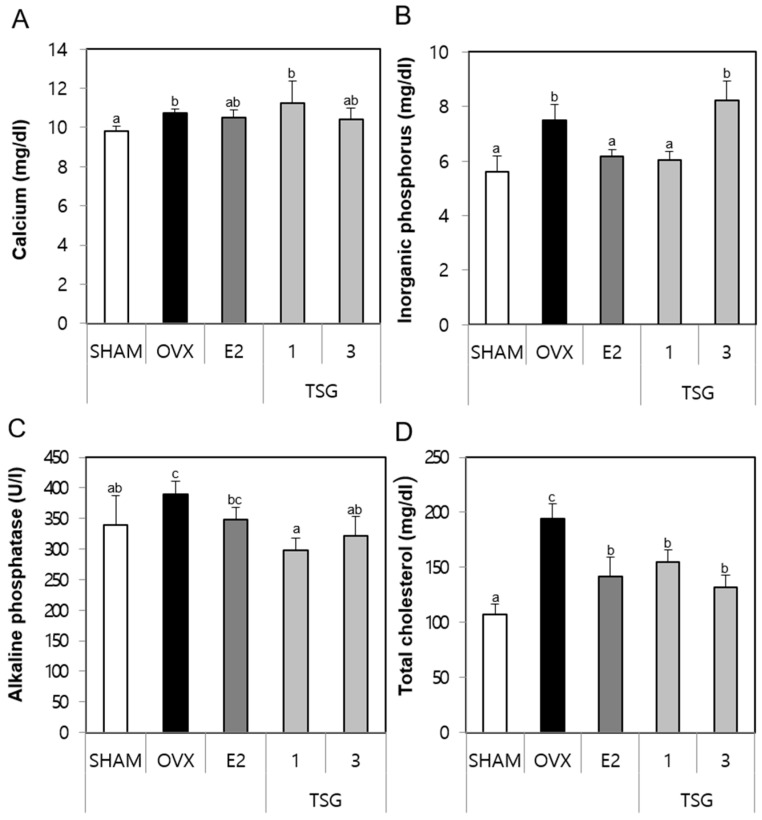
Effect of TSG on the serum biochemical markers. In the control, the SHAM-operated mice and OVX mice with or without the administration of TSG (1 and 3 µg/g/day, I.P) for six weeks, the serum (**A**) calcium, (**B**) phosphorus, (**C**) alkaline phosphatase, and (**D**) total cholesterol were determined using a diagnostic slide. a, b, and c: The means not sharing a common letter are significantly different among the groups at *p* < 0.05 by one-way ANOVA with Duncan’s multiple-range test.

The serum samples obtained from the sacrificed animals were analyzed for the presence of biochemical markers, such Ca, IP, ALP, and TCHO using a diagnostic slide kit. The serum Ca level of the OVX group increased significantly, relative to the SHAM group. Additionally, the serum Ca levels of the OVX+TSG 3 μg/g group was lower than those of the OVX group, but this difference was not significant (Figure 3A). The serum IP level of the OVX group increased significantly, relative to the SHAM group, while that of the OVX+TSG (1 μg/g) group decreased significantly when compared to the OVX group (Figure 3B). The serum ALP of the OVX group was greater than that of the SHAM group, although this increase was not significant. However, the serum ALP level of the OVX+TSG (1 and 3 μg/g) groups decreased significantly, when compared to the OVX group (Figure 3C). Finally, the serum TCHO level of the OVX group increased significantly, relative to the SHAM group (*p* < 0.001), while that of the OVX+TSG (1 and 3 μg/g) groups decreased significantly, relative to the OVX group (Figure 3D). Taken together, these results suggest that TSG affects the bone turnover.

### 2.5. Effect of TSG on Serum Tartrate-Resistant Acid Phosphatase (TRAP) and Bone Specific Alkaline Phosphatase (BALP) in OVX Mice

**Figure 4:**
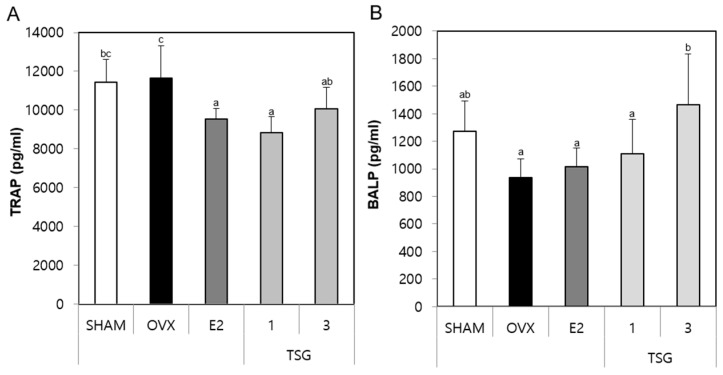
Effect of TSG on (**A**) tartrate-resistant acid phosphatase (TRAP) and (**B**) bone specific alkaline phosphatase (BALP) in the serum after six weeks of treatment. a, b, and c: The means not sharing a common letter are significantly different among groups at *p* < 0.05 by one-way ANOVA with Duncan’s multiple-range test.

The serum samples obtained from the sacrificed animals were analyzed for the biochemical markers, tartrate-resistant acid phosphatase (TRAP) and bone specific alkaline phosphatase (BALP), using an immunoassay (ELISA) kit. The serum TRAP activity of the OVX group tended to increase numerically, although there was no significant increase compared with the SHAM group. However, the serum TRAP level of the OVX+TSG 1 μg/g group decreased significantly, relative to the OVX group (Figure 4A). In addition, the serum BALP level of the OVX group decreased significantly when compared to the SHAM group, while that of the OVX+TSG 3 μg/g group increased significantly, relative to the OVX group (Figure 4B). Thus, these results demonstrate that TSG increases the BALP level and decreases the TRAP activity.

### 2.6. Effect of TSG on Bone Structure Using Micro-Computed Tomography (Micro-CT) in OVX Mice

**Figure 5:**
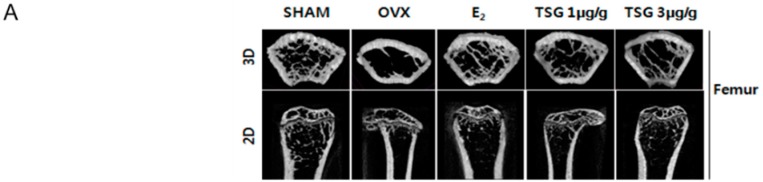

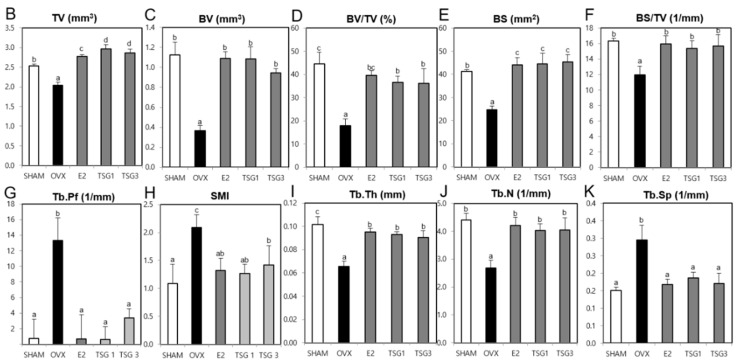
Effect of TSG on the trabecular morphometric parameters in the distal femur of C3H/HeN mice. The mice were treated with vehicle and TSG (1 and 3 µg/g/day, physiological phosphate [IP]) for six weeks. (**A**) The representative two-dimensional (2D) images and three dimensional (3D) images of the femur epiphysis, (**B**) tissue volume, (**C**) bone volume, (**D**) bone volume/tissue volume, (**E**) bone surface, (**F**) bone surface/tissue volume, (**G**) trabecular pattern factor, (**H**) structure model index, (**I**) trabecular thickness, (**J**) trabecular number, and (**K**) trabecular separation, as analyzed by the micro-computed tomography (CT) Skyscan CTAn software. a, b, and c: The means not sharing a common letter are significantly different among groups at *p* < 0.05 by one-way ANOVA with Duncan’s multiple-range test.

**Figure 6:**
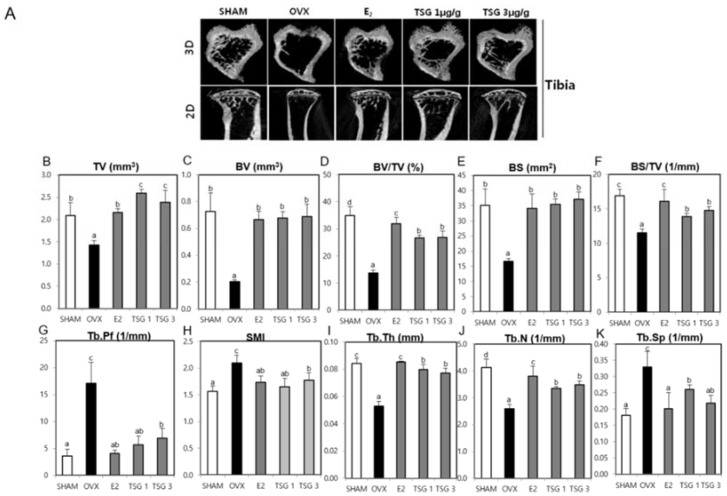
Effect of TSG on trabecular morphometric parameters in the proximal tibia of the C3H/HeN mice. The mice were treated with vehicle and TSG (1 and 3 µg/g/day, IP) for six weeks. (**A**) Representative two-dimensional (2D) images and three dimensional (3D) images of the tibia epiphysis, (**B**) tissue volume (TV), (**C**) bone volume (BV), (**D**) bone volume/tissue volume, (**E**) bone surface, (**F**) bone surface/tissue volume, (**G**) trabecular pattern factor, (**H**) structure model index, (**I**) trabecular thickness, (**J**) trabecular number, and (**K**) trabecular separation as analyzed by the micro-CT Skyscan CTAn software. a, b, c, and d: The means not sharing a common letter are significantly different among groups at *p* < 0.05 by one-way ANOVA with Duncan’s multiple-range test.

Micro-computed tomography (CT) was used to analyze the internal structure of the bones, following TSG treatment, based on the trabecular destruction of the OVX mice. Representative two-dimensional (2D) and three-dimensional (3D) images of the femur and tibia epiphysis of the OVX group showed that the trabecular bone was reduced in comparison with the SHAM group. However, in the OVX+TSG (1 and 3 μg/g) groups, the trabecular was found to be increased relative to the OVX group (Figure 5A and Figure 6A). The tissue volume (TV), bone volume (BV), bone volume/tissue volume (BV/TV), bone surface (BS), bone surface/tissue volume (BS/TV), trabecular thickness (Tb.Th), and trabecular number (Tb.N) of the trabecular morphometric parameters in the OVX group femoral region were significantly decreased, relative to the SHAM group. In contrast, the trabecular pattern factor (Tb.Pf), structure model index (SMI), and trabecular separation (Tb.Sp) of the OVX group tended to increase significantly compared to the SHAM group. The TV, BV, BV/TV, BS, BS/TV, Tb.Th, and Tb.N of the trabecular bone morphology parameters of the OVX+TSG (1 and 3 μg/g) groups were significantly increased, relative to the OVX group, but Tb.Pf, SMI, and Tb.Sp were significantly decreased (Figure 5B–K). The TV, BV, BV/TV, BS, BS/TV, Tb.Th, and Tb.N of the trabecular morphometric parameters in the OVX group tibia region were significantly decreased when compared to the SHAM group, while the Tb.Pf, SMI, and Tb.Sp of the OVX group increased significantly, relative to the SHAM group. The TV, BV, BV/TV, BS, BS/TV, Tb.Th, and Tb.N of the trabecular bone morphology parameters of the OVX+TSG (1 and 3 μg/g) groups were significantly increased when compared to the OVX group, but Tb.Pf, SMI, and Tb.Sp were significantly decreased (Figure 6B–K).

### 2.7. Effect of TSG on Histology of Bones and Uteri of OVX Mice

**Figure 7:**
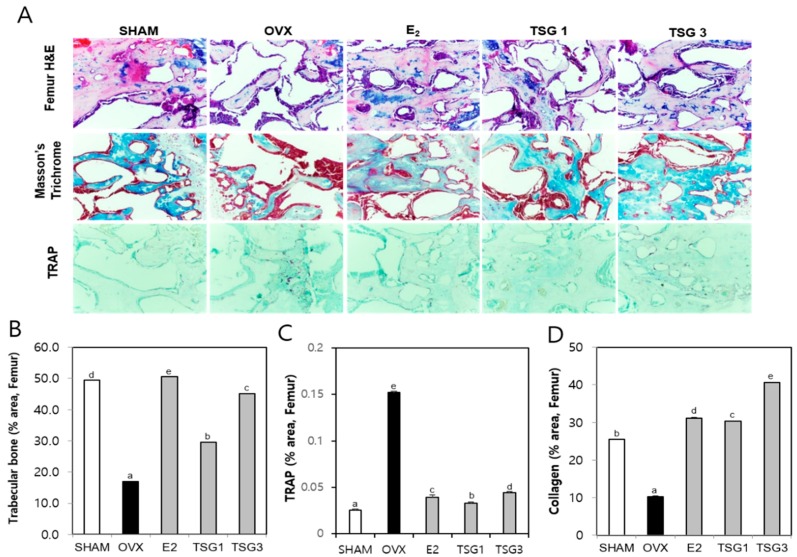
Effect of TSG on the bone tissue of the trabecular in the distal femur of C3H/HeN mice. The mice were treated with vehicle and TSG (1 and 3 µg/g/day, IP) for six weeks. (**A**) Histological analysis of distal femur with hematoxylin and eosin (H and E) and tartrate-resistant acid phosphatase (TRAP), Masson’s trichrome staining (400× magnification); (**B**) trabecular bone area; (**C**) TRAP positive cells and (**D**) collagen in the femur were analyzed using the Image J program. a, b, c, d, and e: The means not sharing a common letter are significantly different among groups at *p* < 0.05 by one-way ANOVA with Duncan’s multiple-range test.

**Figure 8:**
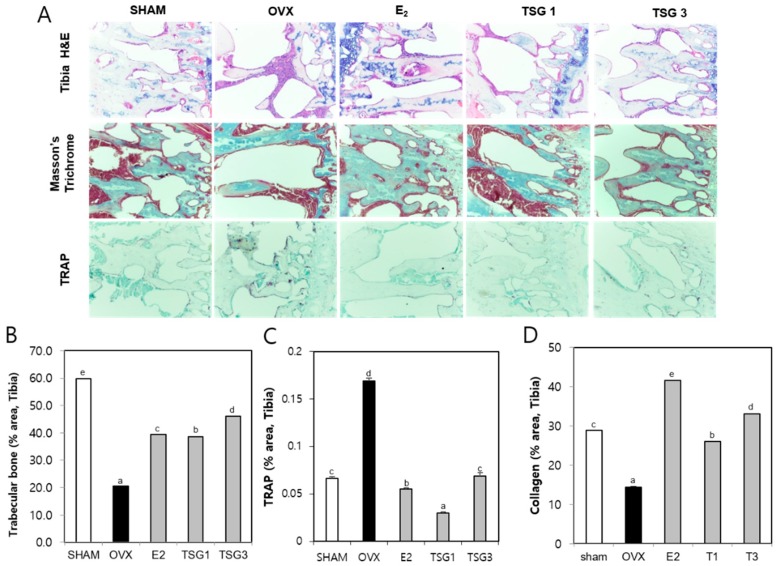
Effect of TSG on the trabecular bone tissue in the proximal tibia of C3H/HeN mice. Mice were treated with vehicle and TSG (1 and 3 µg/g/day, I.P.) for six weeks. (**A**) Histological analysis of the proximal tibia with hematoxylin and eosin (H and E) and tartrate-resistant acid phosphatase (TRAP), Masson’s trichrome staining (400× magnification); (**B**) trabecular bone area; (**C**) TRAP positive cell and (**D**) collagen in tibia were analyzed by the Image J program. a, b, c, d, and e: The means not sharing a common letter are significantly different among groups at *p* < 0.05 by one-way ANOVA with Duncan’s multiple-range test.

Histologic analysis of the distal femur and tibia using hematoxylin and eosin (H and E), TRAP, and Masson’s trichrome staining were performed and described, as shown in Figure 7A and Figure 8A. The area of the femur and tibia trabecular bone of the OVX group was significantly decreased, relative to that of the SHAM group, but those of the OVX+TSG (1 and 3 μg/g) groups were significantly increased, relative to the OVX group (Figure 7B and Figure 8B). The area of the TRAP positive cells in the femur and tibia of the OVX group was significantly increased when compared with the SHAM group. However, in the OVX+TSG (1 and 3 μg/g) group, the area of the TRAP positive cells was significantly lower than that of the OVX group (Figure 7C and Figure 8C). The collagen area of the femur and tibia in the OVX group were significantly decreased, relative to the SHAM group, but those of the OVX and TSG (1 and 3 μg/g) groups were significantly increased, relative to the OVX group (Figure 7D and Figure 8D).

**Figure 9:**
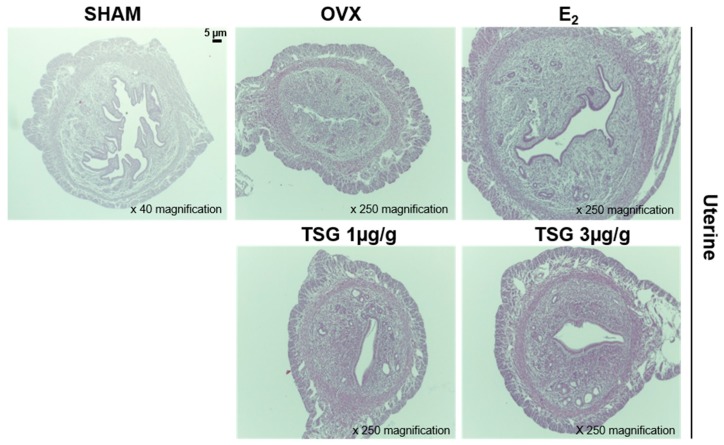
Effect of TSG on uterus tissue in C3H/HeN mice. Mice were treated with vehicle and TSG (1 and 3 μg/g/day, I.P.) for six weeks. Histological changes in the uterus were performed via H and E staining.

The histological analysis of the uterus was performed using H and E staining (Figure 9). The atrophy of the uterine tissue of the OVX group increased relative to the SHAM group, while that of the uterine tissue of the OVS+TSG (1 and 3 μg/g) group decreased when compared to the OVX group.

## 3. Discussion

Osteoporosis, which is a worldwide problem that results in fractures, which lead to disability and high costs to society, affects an estimated 75 million women in Europe, Japan, Australia, and North America [34]. Osteoporosis is also associated with physical limitations, psychosocial impairment, and a reduced quality of life [35]. Many postmenopausal women are treated with hormone replacement therapy (HRT) for osteoporosis, but its long-term use causes side effects, such as breast cancer, venous thromboembolism, coronary heart disease, and stroke [36]. Therefore, the prevention and treatment of osteoporosis using non-hormones or effective and safe alternative compounds are needed.

In many countries, there is growing interest in plant-based treatment for osteoporosis. Traditional Chinese medicines (TCM) contain numerous chemical constituents, which have been widely applied in clinical practice to prevent and treat bone diseases, because they are more suitable for long-term use than synthetic drugs and they have fewer side effects. The natural products contained in TCM have long been regarded as good materials for developing new therapeutic agents [37]. *Polygonum multiflorum* ([PM]; also known as Heshouwu in China), a TCM, exhibits a variety of pharmacological efficacies. In previous studies, we showed that the PM hot water extract contributed significantly to the prevention or treatment of bone loss induced by OVX (ovariectomy) in mice [38]. One major bioactive compound in PM is a 2,3,5,4′-tetrahydroxystilbene-2-*O*-β-d-glucoside (TSG), which possesses anti-oxidative, anti-inflammatory, endothelial protective, and oncogenic enzyme inhibitory activities [39]. However, TSG has not been investigated to determine whether it has anti-osteoporotic effects in OVX-induced mice.

Osteoporosis research uses various animal models such as rodents, rabbits, dogs, and primates. Among these, the laboratory rat is the preferred animal for most researchers [40]. The mouse is a rodent similar to a rat, and is used primarily in osteoporosis animal studies [41,42,43]. In the present study, we investigated the effects of osteoporosis on TSG using the OVX animal model.

The interest in overweight and obesity in postmenopausal women has increased; however, the reasons for increasing the obesity in response to menopause are not clear. Sex hormones influence body fat distribution and adipocyte differentiation. Some researchers have reported that an estrogen deficiency may be an important obesity-triggering factor [44], and several studies have reported that estrogen deficiency increases the body weight of mice [45,46,47]. In the present study, we showed that the administration of TSG effectively inhibited weight gain by estrogen deficiency. These results suggest that TSG may prevent disease associated with increased body weight in postmenopausal women. Moreover, estrogen deficiency induces atrophy of the uterus, and uterine atrophy is evidence of the success of ovariectomy [45]. In the present study, we demonstrated that TSG reduced uterine atrophy. A histological analysis of the uterus showed that OVX induces a reduction of the epithelium thickness, which leads to atrophic histological characteristics. However, the administration of TSG inhibits the reduction of the epithelium thickness. In the OVX mice, the increase in the spleen and thymus weights is associated with the proliferation of T cells, which is known to increase bone loss [48]. In the present study, the spleen and thymus weight were decreased by the treatment of TSG.

The biochemical markers of the bone turnover have been developed over the past 20 years, and are widely applied in the clinical research and clinical trials of new therapies as second endpoints of the treatment efficacy [49]. Calcium (Ca) comprises about 99% of bones and teeth, and therefore affects the bone strength [50]. Maintaining a physiological phosphate (IP) balance also plays an important role in bone health. From the pathophysiology angle, phosphate is one of the main factors involved in the maintenance of bone health, and its deficiency causes clinical illness [51]. Moreover, some studies have reported that total cholesterol (TCHO) is present in high concentrations in postmenopausal women [52]. Total cholesterol is associated with estrogen deficiency. Serum total cholesterol levels increase with estrogen deficiency, and serum total cholesterol was hyperlipidemia in the circulation, leading to increased lipid accumulation in highly vascularized tissues and the arterial wall. The lipoprotein particles entering the blood vessel walls undergo the induction and oxidative changes of various inflammatory processes. As the progenitors of osteoblasts are located adjacent to the subendothelial matrix of the bone vessels, these pathologic processes could be expected to influence the function of these bone-forming cells. As a result, the higher the total cholesterol level is, the more inhibited the formation of osteoblasts and affect bone density, cause to bone loss [53]. In the present study, we showed that TSG suppresses the enhancement of Ca, IP, and TCHO in the OVX mice. These results suggest that TSG inhibits the increase of the bone turnover markers.

Bone is continuously molded, shaped, and repaired through a process termed remodeling, which involves the break down (resorption) and build-up (synthesis) of bone. An imbalance in the bone resorption and bone synthesis by osteoblasts can have a negative impact on the skeletal structure and function, and potentially lead to morbidity and a shortening of lifespan [54]. In general, bone specific alkaline phosphatase (BALP) is a marker of osteoblasts [55], and tartrate resistant acid phosphatase (TRAP) is a well-known marker of osteoclasts [56]. In a previous study, we showed that an estrogen deficiency induced by ovariectomy decreased the BALP and increased the TRAP activity [57]. Additionally, the administration of TSG to OVX mice increased the BALP levels and decreased TRAP. Furthermore, the histological analysis by the TRAP staining of bones showed that TSG inhibited the osteoclast activity. In an in vitro experiment, we confirmed that TSG inhibited osteoclastogenesis (Supplementary 1). Taken together, these results suggest that TSG inhibits a bone remodeling imbalance.

Bone contains calcium, and bone marrow protects important organs and is the site of attachment of muscles and tendons. Macroscopically, bones consist of cortical bone and trabecular bone. Cortical bone comprises ~80% of the skeleton and is found in the femur, tibia, and radius, as well as the outer surfaces of the flat bones and the trabecular bone found mainly at the end of long bones and at the inner parts of the flat bones. Osteoporosis is a disease associated with decreased bone mass, which is characterized by a reduced connectivity, thickness, and number of trabecular bones, which increases the bone fragility and fracture risk. Micro-CT is a technique applied to evaluate the bone structure [58]. In this study, we showed that TSG suppresses the reduction of the cancellous bone thickness and number in the femur and tibia. In contrast, the increase in spacing between the cancellous bone was reduced by TSG administration. These results indicate that TSG may be useful for the treatment of osteoporotic fractures in humans.

## 4. Materials and Methods

### 4.1. Reagents

**Figure 10:**
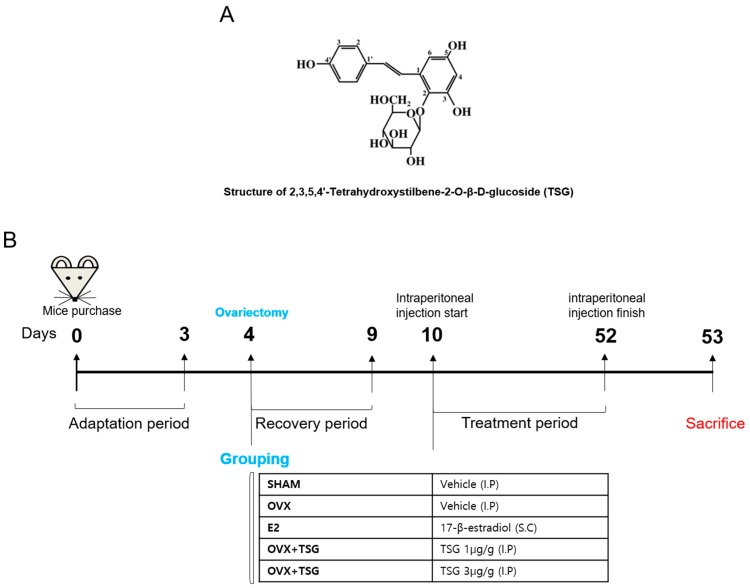
(**A**) The molecular structure of 2,3,5,4’-Tetrahydroxystilbene-2-*O*-β-d-glucoside (TSG); (**B**) experimental protocol for the induction and treatment of osteoporosis, along with the treatment scheme. OVX—ovariectomized mice; E2—estradiol.

2,3,5,4′-Tetrahydroxystilbene-2-*O*-β-d-glucoside (TSG) (Figure 10A) and 17-β-estradiol were purchased from Sigma-Aldrich Co. (St. Louis, MO, USA). All of the materials were dissolved in distilled water and were used.

### 4.2. Osteoporosis Mice Model and Experimental Treatments

There were 25 eight-week-old female C_3_H/HeN mice, weighing 20–22 g, that were purchased from Orientbio (Orientbio Inc., Iksan, Korea). All of the mice underwent a three-day adaptation period before entering the experiment, six weeks during which the mice were housed in standard polycarbonate cages under controlled conditions at 22 °C, 50–60% humidity, and a 12-h light/dark cycle with free access to commercial rodent chow (DAE-HAN Biolink, Daejeon, Korea) and water. Zoletil and Rumpun were used to anesthetize the mice and their ovaries were removed from the dorsal part. After five days of recovery from the surgery, the mice were used for the experiment. All of the animals were managed according to the guidelines of the Animal Protection and Use Committee of Sunchon National University, and all of the procedures were approved by SCNU IACUC (permit number: SCNU IACUC-2018-03, approval’s date: 6 February 2018). A total of 25 mice were divided into five groups. A model of osteoporosis was attained by conducting ovariectomy surgery on female mice. Six days after surgery, the mice were randomly divided into five groups (*n* = 5 for each group), and were intraperitoneally treated with TSG for six weeks, as follows: a sham group (sham surgery with no treatment), an ovariectomy control group (OVX, ovariectomy with no treatment), estradiol treatment group (E2, 0.03 µg/head/day, s.c.), a low dose TSG treatment group (TSG 1 µg/g/day), and a high dose TSG treatment group (TSG 3 µg/g/day). β-estradiol and TSG were administered for six weeks, during which time the body weight was recorded weekly. The day after the last administration of E2 and TSG, the animals were sacrificed by cervical dislocation, and the serum, uterus, spleen, thymus, femur, and tibia were obtained. The serum samples were stored at −80 °C until analysis. The femur and tibia were weighed and their lengths determined using a Vernier caliper. The TSG treatment experiment plan is summarized in Figure 10B.

### 4.3. Analyses of Serum Ca, IP, ALP, and TCHO

The blood was obtained from the orbital venous part of the anesthetized mice prior to being sacrificed, and was then centrifuged at 5000 rpm for 5 min. The supernatant was then collected and stored at −80 °C until being analyzed for serum calcium (Ca), inorganic phosphorus (IP), alkaline phosphatase (ALP), and total cholesterol (TCHO) levels. All of the samples were analyzed using an automatic analyzer (Dri-Chem 3500i, Fujifilm Medical System Co., Ltd., Tokyo, Japan) and a diagnostic slide kit.

### 4.4. Analyses of Serum Tartrate-Resistant Acid Phosphatase (TRAP) and BALP

A TRAP enzyme-linked immunoassay (ELISA) kit (USCN Life Science, Wuhan, China) was used to determine the activity of tartrate-resistant acid phosphatase (TRAP), a bone resorption marker. The level of bone alkaline phosphatase (BALP), an osteogenic marker, was measured using a BALP ELISA Kit (Mybiosource, San Diego, CA, USA).

### 4.5. Measurement of Bone Structure Using Micro-Computed Tomography (CT)

Analyses were conducted as previously described [59]. Briefly, the morphometric parameters of the bones (femur and tibia) were determined using a micro-computed tomography (micro-CT) system (Skyscan 1272, Bruker micro-CT, Kontich, Belgium). Specifically, the tissue volume (TV), bone volume (BV), bone volume/tissue volume (BV/TV), bone surface (BS), bone surface/tissue volume (BS/TV), trabecular thickness (Tb.Th), trabecular separation (Tb.Sp), trabecular pattern factor (Tb.Pf), structure model indices (SMIs), and trabecular number (Tb.N) were evaluated. The results were visualized using two-dimensional (2D) and three-dimensional (3D) images, and the CTAn (CT-Analyser) software (Skyscan, Kontich, Belgium) was used to analyze the structural parameters of the trabecular bone. In addition, the CTvol software was used to regenerate 3D images of the trabecular bone. The specifications of the micro-computed tomography (micro-CT) system (SkyScan 1272, Bruker micro-CT, Kontich, Belgium) were as follows: bone scans were taken with a source voltage of 70 kV and a source current of 142 μA. The resolution was set to 13.27 μm and the rotation step was 0.2°. The image reconstruction was performed using the NRecon software (1.1.9, SkyScan, Kontich, Belgium).

### 4.6. Bone and Uterus Histological Analysis

The bone and uterus staining were performed as previously described [59,60]. Briefly, the bone samples were fixed in 4% formaldehyde at room temperature, and decalcified in 10% ethylenediaminetetraacetic acid (EDTA). The samples were then dehydrated, embedded in paraffin, sectioned at 5 μm, and stained with hematoxylin and eosin (H and E). Next, the samples were stained with TRAP reagent to measure the osteoclast activity. To accomplish this, 225 μM naphthol AS-MX phosphate (Sigma-Aldrich), 0.84% *N*,*N*-dimethylformamide (Sigma-Aldrich), and 1.33 mM Fast Red Violet LB Salt (Sigma-Aldrich) in 50 mM sodium acetate (pH 5.0) containing 50 mM sodium tartrate were applied to the sections. The samples were then washed in distilled water and counterstained with 1% methyl green. The sections were also deplasticized in 2-ethoxyethyl acetate and stained with Masson’s trichrome [61]. The image J program (National Institutes of Health, Bethesda, MD, USA) was then used to analyze the trabecular bone, TRAP positive cells, and collagen. The trabecular area % was calculated based on the ratio of the trabecular bone area to the total bone area. The measured concentration of the TRAP positive cells (TRAP % area) was quantified relative to the total trabecular bone surface. The collagen area % was calculated based on the ratio of the collagen area to the total bone area. Before the uterus dries, the uteri were fixed in a 4% neutral-buffered formalin for 24 h at room temperature. The paraffin-embedded cross sections were cut and mounted on saline-coated glass slides.

### 4.7. Statistical Analysis

The data are presented as the means ± standard deviations (SDs). Statistically significant differences between groups were identified by one-way analysis of variance (ANOVA) using SPSS version 22 (Chicago, IL, USA) with Duncan’s multiple range test. In addition, *p* < 0.05 was considered to indicate statistical significance.

## 5. Conclusions

Estrogen deficiency in ovariectomized mice results in decreased bone formation, increased bone resorption, and fibrous bone loss. Therefore, we measured the biochemical markers that assess bone formation and bone resorption through the serum of mice. We also performed a micro-CT and histological analysis measuring the internal structure of bone to determine whether TSG inhibits bone loss as a result of estrogen deficiency. This study demonstrated for the first time that the administration of TSG suppresses the destruction of the bone microarchitecture through a reduction of TRAP activity and increased bone turnover markers, such as Ca, IP, ALP, and TCHO. From a therapeutic point of view, TSG is a good candidate material for treating or preventing osteoporosis and complications in postmenopausal women.

## Figures and Tables

**Table 1:** The effect of 2,3,5,4′-tetrahydroxystilbene-2-*O*-β-d-glucoside (TSG) on the thymus and spleen weight in ovariectomized (OVX) mice.

	SHAM	OVX	E2	TSG1	TSG3
**Thymus weight (mg/g BW)**	0.97 ± 0.27 ^a^	1.2 ± 0.03 ^b^	0.95 ± 0.08 ^a^	1.01 ±0.09 ^a^	1.07 ± 0.07 ^a,b^
**Spleen Weight (mg/g BW)**	3.27 ± 0.11 ^c,d^	3.29 ± 0.16 ^d^	2.79 ± 0.12 ^a^	2.93 ± 0.13 ^a,b^	3.08 ±0.28 ^b,c^

TSG 1 µg/g and TSG 3 µg/g decrease the OVX-induced increase in the thymus and spleen weight. ^a^, ^b^, ^c^, and ^d^: The means not sharing a common letter are significantly different among the group at *p* < 0.05 by one-way analysis of variance (ANOVA) with Duncan’s multiple-range test. E2—estradiol.

**Table 2:** The effect of TSG on the bone weight and length in OVX mice.

	Length (mm)		Weight (mg)	
	Tibia	Femur	Tibia	Femur
**SHAM**	16.6 ± 0.33 ^b^	1.94 ± 0.75 ^a,b^	55.46 ± 4.79 ^b,c^	44.9 ± 2.87 ^b,c^
**OVX**	15.98 ± 0.27 ^a^	19.05 ± 0.37 ^a^	45 ± 2.70 ^a^	40.7 ± 1.70 ^a^
**E_2_**	17.08 ± 0.25 ^c^	19.8 ± 0.59 ^b^	59.24 ± 1.76 ^c,d^	46.62 ± 0.62 ^c^
**TSG1**	16.52 ± 0.18 ^b^	19.33 ± 0.2 ^a,b^	54.8 ± 2.11 ^b^	43.1 ± 1.59 ^a,b^
**TSG2**	16.52 ± 0.13 ^b^	19.28 ± 0.18 ^a,b^	5.98 ± 2.33 ^d^	44.08 ± 1.64 ^b,c^

TSG 1 µg/g and TSG 3 µg/g increase the OVX-induced decrease in the weight and length of bone. ^a^, ^b^, ^c^, and ^d^: The means not sharing a common letter are significantly different among group at *p* < 0.05 by one-way ANOVA with Duncan’s multiple-range test.

## Supplementary Materials

Supplementary materials can be found at http://www.mdpi.com/1422-0067/19/9/2554/s1.
