# Supplementary material for: Protective Effects of 2,3,5,4′-Tetrahydroxystilbene-2-O-β-d-glucoside on Ovariectomy Induced Osteoporosis Mouse Model
Source: Int J Mol Sci. 2018 Aug 28;19(9):2554. doi: 10.3390/ijms19092554 (PMC6163345; doi:10.3390/ijms19092554)
Supplement: Supplementary file 1 [file ijms-19-02554-s001.zip › ijms-334108-SI.pptx]

## Slide 1
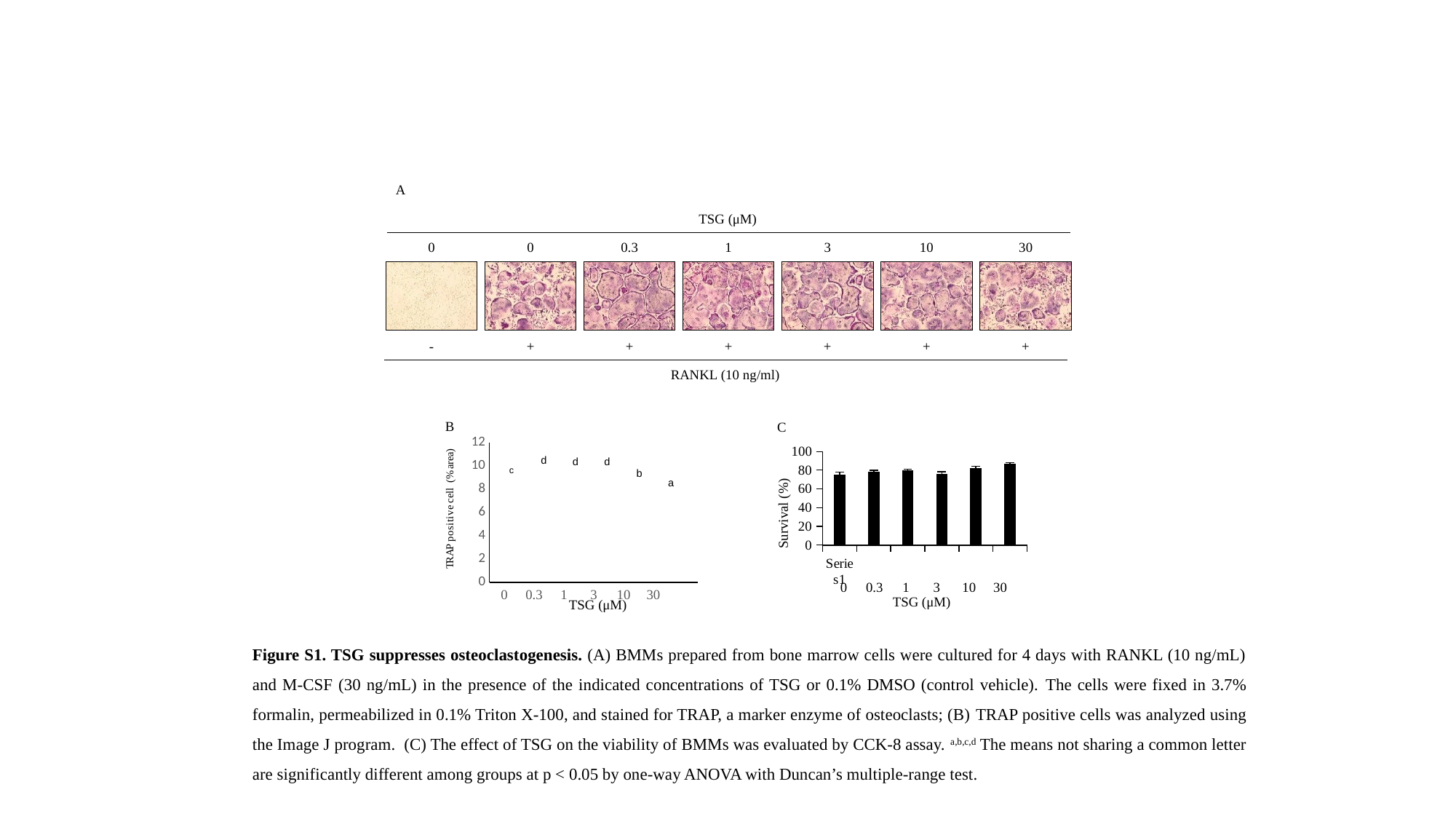

A
TSG (μM)
0
0
0.3
1
3
10
30
-
+
+
+
+
+
+
RANKL (10 ng/ml)
B
C
### Chart
| Category | |
|---|---|
| 0 | 0.6240089171170792 |
| 0.3 | 0.6604009490928208 |
| 1 | 0.6645406226271829 |
| 3 | 0.6570482972701542 |
| 10 | 0.5890261234552382 |
| 30 | 0.5273860891467267 |
### Chart
| Category | |
|---|---|
| | 74.99999999999999 |
| | 78.07240803263697 |
| | 79.27771505943588 |
| | 75.32033832735455 |
| | 81.51422592670745 |
| | 86.51033973412112 |d
d
d
c
b
a
Survival (%)
0
0.3
1
3
10
30
TSG (μM)
TSG (μM)
Figure S1. TSG suppresses osteoclastogenesis. (A) BMMs prepared from bone marrow cells were cultured for 4 days with RANKL (10 ng/mL) and M-CSF (30 ng/mL) in the presence of the indicated concentrations of TSG or 0.1% DMSO (control vehicle). The cells were fixed in 3.7% formalin, permeabilized in 0.1% Triton X-100, and stained for TRAP, a marker enzyme of osteoclasts; (B) TRAP positive cells was analyzed using the Image J program. (C) The effect of TSG on the viability of BMMs was evaluated by CCK-8 assay. a,b,c,d The means not sharing a common letter are significantly different among groups at p < 0.05 by one-way ANOVA with Duncan’s multiple-range test.
